# Perinatal Infection: A Major Contributor to Efficacy of Cooling in Newborns Following Birth Asphyxia

**DOI:** 10.3390/ijms22020707

**Published:** 2021-01-12

**Authors:** Jibrin Danladi, Hemmen Sabir

**Affiliations:** 1 Department of Neonatology and Pediatric Intensive Care, Children’s Hospital University of Bonn, 53127 Bonn, Germany; hemmen.sabir@ukbonn.de; 2 German Center for Neurodegenerative Diseases (DZNE), 53127 Bonn, Germany

**Keywords:** newborn, hypoxic-ischemic encephalopathy, therapeutic hypothermia, infection, cold shock proteins, heat shock proteins

## Abstract

Neonatal encephalopathy (NE) is a global burden, as more than 90% of NE occurs in low- and middle-income countries (LMICs). Perinatal infection seems to limit the neuroprotective efficacy of therapeutic hypothermia. Efforts made to use therapeutic hypothermia in LMICs treating NE has led to increased neonatal mortality rates. The heat shock and cold shock protein responses are essential for survival against a wide range of stressors during which organisms raise their core body temperature and temporarily subject themselves to thermal and cold stress in the face of infection. The characteristic increase and decrease in core body temperature activates and utilizes elements of the heat shock and cold shock response pathways to modify cytokine and chemokine gene expression, cellular signaling, and immune cell mobilization to sites of inflammation, infection, and injury. Hypothermia stimulates microglia to secret cold-inducible RNA-binding protein (CIRP), which triggers NF-κB, controlling multiple inflammatory pathways, including nod-like receptor family pyrin domain containing 3 (NLRP3) inflammasomes and cyclooxygenase-2 (COX-2) signaling. Brain responses through changes in heat shock protein and cold shock protein transcription and gene-expression following fever range and hyperthermia may be new promising potential therapeutic targets.

## 1. Introduction

Neonatal asphyxia describes a condition in newborns due to deprivation of blood carrying oxygen and nutrients (hypoxia-ischemia, HI) from the placenta to the fetus before or during delivery. Hypoxic-ischemic encephalopathy (HIE) is the feared neurological consequence that may occur in newborns following neonatal asphyxia causing brain inflammation and immunodepression [1]. Neonatal encephalopathy (NE) occurs in one to three of every 1000 births in high-income countries (HICs) [2,3] and approximately 10 to 20 of every 1000 births in low- and middle-income countries (LMICs) [4]. It is estimated that approximately 10% of the affected newborns die in their postnatal age, 25% develop severe and permanent neurological disabilities [5] such as cerebral palsy, seizures, mental retardation, learning impairment, and epilepsy [6,7,8,9]. Global statistics have shown that about 99% of annual neonatal deaths occur in the LMICs, and 1% in HICs [10].

For decades, multiple pre-clinical studies have been employed either using animal models of global or focal HI or cell culture models of oxygen-glucose deprivation, investigating the ameliorating effects of many chemical compounds on neuronal lesions. Recent pre-clinical and clinical research has shown that certain compounds have neuroprotective effects, suggesting that their use could be generalized for clinical practice in the near future. Additionally, the application of therapeutic hypothermia immediately after the hypoxic-ischemic event could prolong the window of opportunity for pharmacological therapeutic interventions. Therapeutic hypothermia is the standard treatment for NE of presumed HI origin in the HIC [11]. The controversy among physicians remains on whether hypothermia can also be administered safely and provide neuroprotection in other diseases, like traumatic brain injury or metabolic diseases. Therapeutic hypothermia has demonstrated efficacy in preventing perinatal brain injury following HIE [12]. There are several clinical trials and meta-analyses of newborns with HIE [13,14], showing neuroprotection from therapeutic hypothermia in HIC. However, it is not clear if therapeutic hypothermia is neuroprotective following birth asphyxia in LMIC. Emerging data have shown that therapeutic hypothermia is not safe for neonates in LMIC [15]. Also, the decrease in neonatal body temperature below 35 °C (accidental hypothermia) is the major cause of mortality in the LMIC. It has been shown experimentally that perinatal infection limits the neuroprotective effect of therapeutic hypothermia and clinically enhances neurotoxicity, and increases mortality in term asphyxiated neonates in LMIC.

Our review will focus on the features of changes in body temperature that often accompanies infections and inflammation acting as a biological response modifier by regulating signaling pathways and gene expression involved in immune defense, inflammation, cell death, and survival. We discuss the etiology of perinatal infection, the potential benefit of hypothermia, and how elements of the heat shock (HS) and cold shock (CS) response pathways affect hypothermia’s success over immune response modifiers.

## 2. Perinatal Infection and Hypoxic-Ischemic Encephalopathy

**Figure 1:**
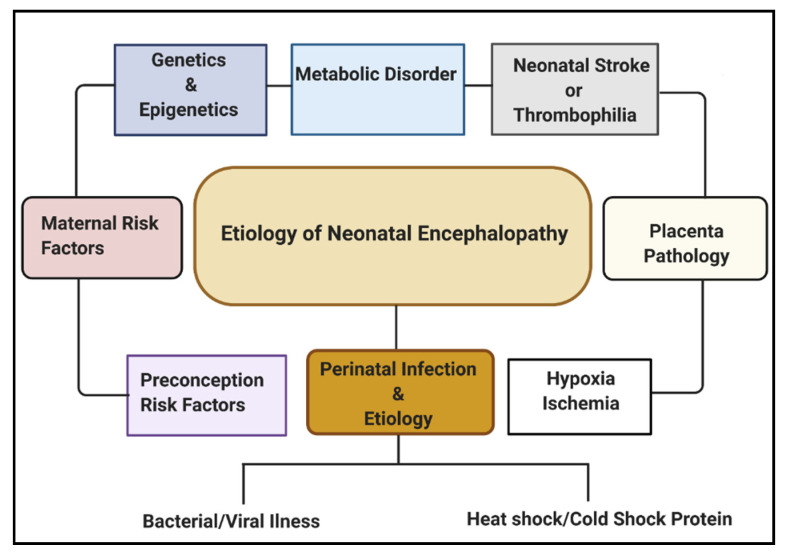
Etiologies involved in neonatal encephalopathy.

Multiple etiological factors predisposing to NE have been described. They include antenatal maternal factors, hypoxia-ischemia, placental pathologies, neonatal stroke/thrombophilia, genetics and epigenetics, metabolic disorders, and perinatal infection (Figure 1). Infections during pregnancy can increase the expression of cytokines, causing inflammation to the fetal brain, leading to brain damage in the fetus and, subsequently, the newborn [16]. Some types of infection that have been linked with neonatal brain injury include viruses such as chickenpox, rubella, cytomegalovirus (CMV), and bacterial infections such as infections of the placenta or fetal membranes, or maternal pelvic infections.

Regarding global neonatal mortality, a prospective study by Karsten et al. [17] reported that the global rate of severe infections accounted for 36% of all neonatal deaths, 29% were due to prematurity, and 23% were due to birth asphyxia. Evidence from recent studies has shown that HI brain damage plus perinatal intrauterine infection are the major etiological risk factors for cerebral palsy (CP). Both in vivo and in vitro neurologic assessments reveal that term infants born to mothers with clinical chorioamnionitis, suffered from a inflammatory cytokine storm, which is correlated with CNS abnormalities [18]. Also, several data have shown that both infection and HI in the brain promotes the production of proinflammatory cytokines, which may lead to further injury in the cerebral tissue [19,20,21].

Studies have shown that severe infection and prematurity-associated mortality is increased due to mild (33–36 °C) and moderate (28–32 °C) hypothermia [22]. Perinatal infection pre-sensitizes the fetal brain and makes it vulnerable to HI [23,24]. A previous study showed that therapeutic hypothermia was not neuroprotective in a LPS-sensitized HI brain injury model in newborn rats [25]. The authors observed a dramatic loss of brain area after inflammation-sensitized HI brain injury, which was not reduced by therapeutic hypothermia.

## 3. Mechanisms and Pathways of Perinatal Infection

**Figure 2:**
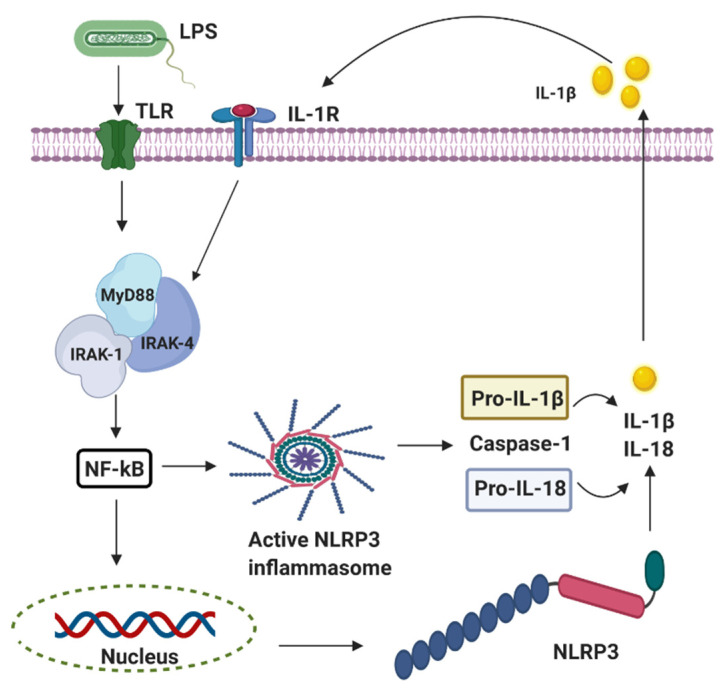
The activation of nod-like receptor (NLR) family pyrin domain containing 3 (NLRP3) inflammasomes involves multiple endogenous or exogenous stimuli. LPS stimulation stimulates Toll-like receptors (TLRs), which lead to the up-regulation of NLRP3, IL-1 via nuclear factor kappa B (NF-κB) dependant Myeloid differentiation factor 88 (MyD88), IL-1 receptor-associated kinase 1/4 (IRAK4).

Perinatal infection stimulates the innate immune and inflammatory responses, causing the intracellular production of pro-interleukin 1 beta (IL-1β) following the stimulation of the pattern-recognition receptors (PRRs) such as the Toll-like receptors (TLRs) and leading to subsequent cell death [26]. The innate branch of the immune system relies heavily on TLRs and Nod-like receptors (NLRs) to detect and dissemble invading pathogens. Most of the important cell types expressing TLRs are the antigen-presenting cells (APCs), including macrophages, dendritic cells, and B lymphocytes [27]. Experimentally, TLRs are identified in most cell types and can be expressed either constitutively or inducible during infection [28,29,30]. The expression of TLRs and NLRs on and in both migrating and non-migrating cells is crucial for the rapid response to foreign invaders. The severity of a perinatal infection-induced inflammatory storm in neonates is strongly associated with nuclear factor kappa B (NF-κB) activation. It leads to nod-like receptor family pyrin domain containing 3 (NLRP3) inflammasome activation, followed by an increase of IL-1β expression, up-regulation of cyclooxygenase-2 (COX-2), and transient receptor potentials (TRPs). The up-regulation of COX-2 and TRPs acting as both sensor and effector shuffling among the nervous, vascular, and immune system will be discussed later. The activation of functional pro-IL-1β requires proteolytic cleavage, predominantly by caspase-1, and a component of the NLRP3 inflammasome multi-protein complex, resulting in secretion of mature biologically active IL-1β (Figure 2) [17]. Activation of the NLRP3 inflammasome triggers local mediators of the host cell damage in vivo, such as free radicals and DNA or adenosine triphosphate (ATP).

## 4. Cyclooxygenase 2 (COX-2)-Induced Fever in Perinatal Infection Following Birth Asphyxia 

**Figure 3:**
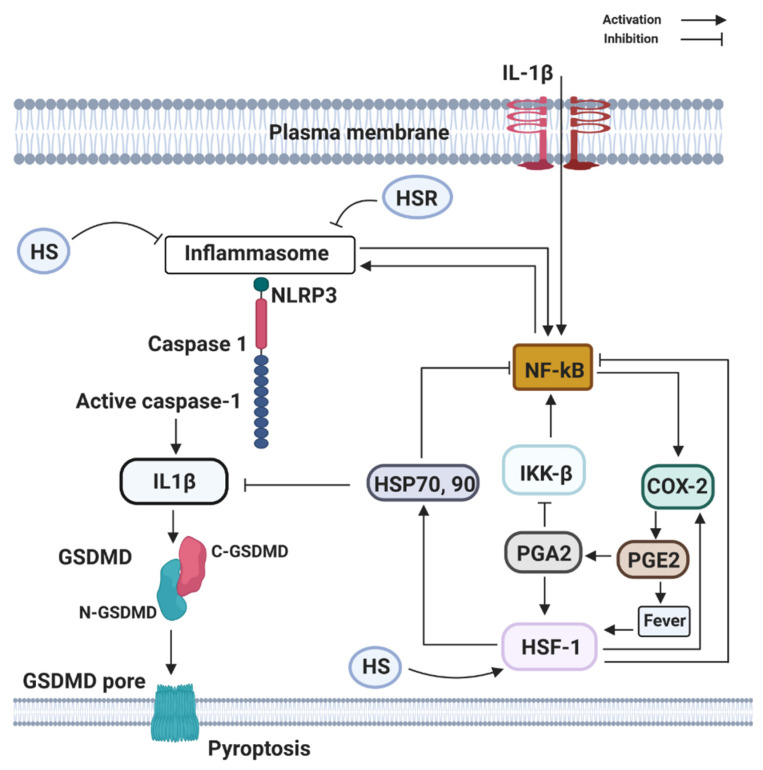
Components of the heat shock response (HSR) (HSP70 and HSF1) can directly or indirectly block the activation and transcribing activity of NF-κB. HSR is mainly centered on the heat shock transcription factor-1 (HSF1) that leads to the large production of the 70 kDa family of heat shock proteins (HSP70, 90). HSF1 may be directly activated by PGE2-induced rise in temperature (fever), by heat shock (HS), by estrogen (E2), and PGE2-derivative PGA2, whose physiological production is enhanced at late stages of inflammation. Activation of NLRP3, recruit the inflammasome adaptor ASC, which engages caspase-1. Subsequently, caspase-1 cleaves precursor IL-1β and IL-18 to their bioactive fragments, and also Gasdermin D (GSDMD) to trigger GSDMD N-domain pore formation in the plasma membrane. The GSDMD pores allow efficient IL-1β, eventually, cause the lytic cell death known as pyroptosis.

COX-2 is an eicosanoid that generates arachidonic acid-derived lipid autacoids, including prostaglandins (PGs), thromboxanes, and leukotrienes. COX-2 is a critical mediator of inflammation, resolution, and tissue homeostasis. It is involved in a broad range of physiological processes, such as inflammation, fever, allergy, and pain. The presence of infection triggers the upregulation of inositol-requiring enzyme 1α–X-box binding protein 1, a branch of the endoplasmic reticulum (ER) stress pathway, to direct the expression of microsomal prostaglandin E synthase-1 and prostaglandin-endoperoxide synthase 2 (COX-2), which mediate the biosynthesis of prostaglandins (PGE2, PGD2, and PGF2α) from arachidonic acid. COX-2 production is associated with the production of a cytokine storm. Accordingly, ER stress signaling alone can cause a slight increase in the level of IL-6 (Figure 3). All together, in the presence of PGE2, interferon-γ, and activated endoplasmic reticulum stress, IL-6 production is greatly enhanced in glial cells. However, the key role of COX-2 in neonatal asphyxia inflammation and resolution has not been fully understood; studies have shown that cytokine overload in neonatal asphyxia contributes largely to morbidity and mortality [31]. As increased proinflammatory cytokines remain the driving force in severe neonatal asphyxia, the up-regulation activity of COX-2 following neonatal asphyxia may regulate the cytokine storm through fever induction. Fever is one of the usual clinical features that appear during the course of several infectious diseases. Fever is a process in which the body temperature rises, deviating from normal values, and according to Saladin and Porth [32], fever is a beneficial process as long as it does not persist or reaches 44 °C to 46 °C, where it could be fatal or lead to irreversible brain damage. Fever has been demonstrated to affect other immune cells as reflected by Harden et al. [33,34], including different types of innate immune cells such as neutrophils, monocytes, and T-cells or Natural Killer cells (NK) [35]. 

Fever has been suggested to be an essential product of several biological processes, where the detection of unchained pathogens sets up events that end up in favor of the host [36]. It is of paramount importance to understand the mechanisms of infection, where potential effects of fever on this process may have been overlooked. The induction of fever during neonatal asphyxia is associated with COX-2 expression, and the exposure of humans and rodents to temperatures ranged between 41–43 °C can induce heat shock response (HSR), leading to induction of heat shock protein (HSP) synthesis [37]. HSR is connected with immune responses and attenuates cytokines release [37], important for the host immune response and pathogen mechanisms of evasion. Pathogen-induced overexpression of HSPs is fundamental for the survival of the host organism during macrophage infection [38,39,40].

## 5. Heat Shock Proteins Stabilize Correct Protein Folding during Fever Following Birth Asphyxia

Heat shock proteins (HSPs) are present in all organisms and cell types. They are phylogenetically conserved proteins having both structural and functional significance, and they can be stimulated by stress signals (e.g., heat shock) and pathophysiological states (e.g., fever, inflammation, and infection) as well as those induced by normal development stress [41,42,43]. It has been shown that increased core body temperature has a protective role in the outcome of infection. Ostberg et al. [44] and others Jiang et al. [45] showed that mild systemic heating at 39.5 °C enhances the concentration of tumor necrosis factor alpha (TNF-α) and IL-6 in the blood and tissues of mice sensitized with bacteria endotoxin LPS. Heat shock proteins’ function in protein folding prevents protein denaturation or cell death under stressful conditions [46,47]. Although HSPs are intracellular proteins, they can be recruited to the plasma membrane or released into the extracellular environment and have immunomodulatory functions [48]. Most HSPs are involved in the synthesis and release of proteins from various cells either during cell injury or during translocation to the plasma membrane and are then secreted [49].

HSPs are known to have both positive and negative effects in regulating macrophage function, and this may depend on the cellular location of these HSPs. It is proposed that extracellular HSPs might serve as a danger signal to the immune response, whereas intracellular HSPs could serve as a negative regulator to control the inflammation [50]. Previous studies have shown that extracellular HSPs exert immune-stimulatory effects [48]. Wang et al. [51] demonstrated that extracellular HSP70 binds to lipid raft microdomains on the plasma membrane of macrophages and enhances their phagocytic ability. In fact, HSP70-mediated phagocytosis is very crucial for the internalization of antigens to CD4+ T-cells. It is clear that extracellular HSPs can largely stimulate the release of TNF-α, IL-6, IL-1β, IL-12, and nitrous oxide (NO). As well, chemokines are released by monocytes/macrophages [52,53,54,55], orchestrated through the CD14/TLR (both TLR2 and TLR4) complexes-activating downstream NF-κB and mitogen-activated protein kinase (MAPK) pathway [56,57,58]. In addition, HSPs also assists in the trafficking and targeting complex toward the Golgi apparatus [59]. This indicates that the elevation of extracellular HSPs may serve as endogenous danger signals to alert the host defense system through their cytokine-like function. HSPs have been studied for their potential to protect the brain from ischemic injury. They protect from both global and focal ischemia in vivo, and cell culture models of ischemia/reperfusion injury in vitro [60]; however, the mechanism of protection is not well understood. Although several members of the HSPs have shown to function as anti-apoptosis after HI brain injury, overexpression of HSP70 prevents the release of cytochrome c from mitochondria and the activation of casepase-9 by binding to apoptotic protease activating factor 1 (Apaf-1), thus blocking the caspase-dependent apoptotic pathway [61]. HSP90 binds to phosphorylated protein kinase B (Akt/PKB) and promotes the phosphorylation of the pro-apoptotic proteins Bax and caspase-9, and blocks the mitochondrial apoptosis pathway [61]. 

One member of the HSP90 family is glucose-regulated protein 94 (GRP94). Its expression has been shown to inhibit the activation of caspase-3 and calpain, maintaining the intracellular calcium homeostasis to protect neurons [61].

## 6. The Role of TRPV1 in Perinatal Infection Following Birth Asphyxia 

Transient receptor potential vanilloid channel 1 (TRPV1) is highly expressed in high temperate nerve fibers and is activated by heat, protons, and both endogenous and exogenous agonists [62,63]. TRPV1 is a nonselective cation channel that plays a significant role in thermoregulation, although the exact mechanisms in thermal regulation have yet to be fully understood [62,64,65]. Intravenous infusion of dihydrocapsaicin (DHC), a chili-derived TRPV1 agonist, and other capsaicinoids have shown the capacity to produce hypothermia in rodents and larger mammals [66]. In contrast, perinatal infection-induced hyperthermia was shown to occur through the TRPV1 channel [67,68,69,70]. It appeared that TRPV1 antagonists cause hyperthermia by blocking the tonic suppression of the autonomic cold defenses: thermogenesis and skin vasoconstriction [67,71]. In the brain, TRPV1 mediates cellular processes such as synaptic transmission, neurogenesis, and neuroinflammation [72,73]. Recently, TRPV1 has gained attraction from its functional expression in microglia and astrocytes [74,75]. It stimulates janus kinase 2- signal transducer and activator of transcription 3 (JAK2-STAT3) to regulate astrocyte and microglial activation and the expression of IL-1β and IL-6 [76,77,78,79]. TRPV1 deficiency in microglia and astrocytes has been shown to attenuate the expression of ionized calcium-binding adapter molecule 1 (Iba1), glial fibrillary acidic protein (GFAP), and IL-1β by reducing phosphorylation of NF-κB, JAK2, and STAT3. As well, a decrease in IL-1β is associated with TRPV1 deficiency by inhibiting activation of NLRP3 inflammasome. Not much is known about the role of TRPV1 in perinatal infection and birth asphyxia. However, in a selective study of the TRPV1 receptor, it was evident that TRPV1 expression is associated with a protective effect in the onset of sepsis after endotoxin [80]. Additionally, neonatal HI-induced neuro-behavioral disorders were significantly improved in mice lacking TRPV1 [81].

## 7. The Role of Cold Shock Protein in Hypothermia Following Birth Asphyxia

**Figure 4:**
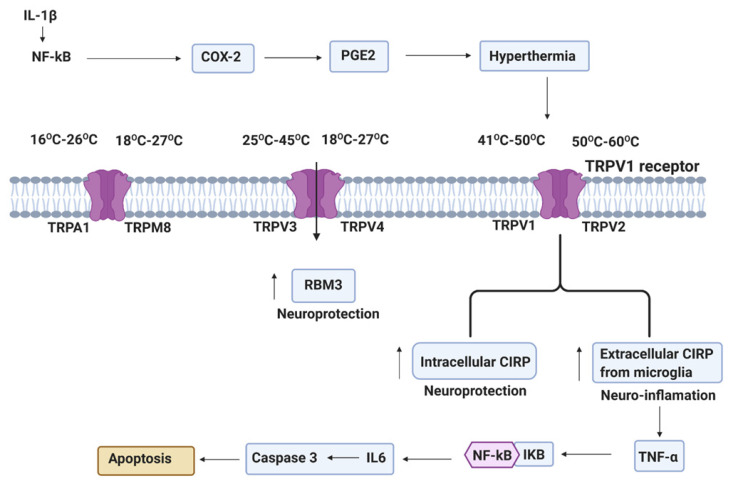
Systemic inflammation from perinatal insults can induce COX2 in reactive glia cells (astrocytes and microglia). PGE2 production after COX2 activation leads to EP1 receptor mediated maturation arrest of oligodendrocyte progenitor cells (OPCs). TRPs plays an important role in a wide range of temperature increase and stimulate hypothermia. Hypothermia-induced neuroprotection via RNA-binding motif protein 3 (RBM3) and intracellular cold-inducible RNA-binding protein (CIRP), and extracellular CIRP-induced deleterious inflammation via NLRP3 inflammasome (NLRP3, ASC, and caspase-1), which mediate the activation of caspase-1 and then stimulated the cleavage of pro-IL-1β [81].

The two described CSPs in mammals are cold-inducible RNA-binding protein (CIRP) and RNA-binding motif protein 3 (RBM3). The CIRP provides neuroprotection via its intracellular activity, while its extracellular activity is detrimental in enhancing the inflammatory response. Interest in RBM3 has significantly increased due to its critical role in the protective effect of hypothermia (Figure 4). The physiology of the therapeutic effects of hypothermia provides key protective targets for reducing ischemic brain injury [82]. It is well known that the transcription and protein levels of CIRP, RBM3, and splicing factor arginine/serine-rich 5 (SRSF5) are affected by mild hypothermia [83,84,85]. The transcription factor Sp1 and the promoters in the CIRP gene are critical in the enhancement of splicing efficiency in the induction of CIRP [86,87,88]. However, the mechanisms of temperature sensing and the signaling pathways by which hypothermia induces the expression of cold-inducible proteins (CIPs) are partially understood. A study using antagonists in the presence of shRNA against TRPV4 demonstrated that TRPV4 is required for the induction of CIPs [85]. Hypothermia has been shown to down-regulates global protein synthesis and cell metabolism. It also up-regulates cold shock proteins (CSPs).

RMB3 is a glycine-rich protein that promotes global protein synthesis at 32 °C by accelerating ribosome assembly, stabilizing mRNA and decreasing microRNA expression. In perinatal asphyxia models, RBM3 mediates the rescue from apoptotic neuronal death during therapeutic cooling [89]. Up-regulated RBM3 expression is associated with hibernation; it helps restore brain activity in awakening animals and protects cells against cold damage. RBM3 has been shown to stimulate neuronal differentiation and inhibit HI-induced apoptosis in the two main areas of persistent adult neurogenesis, the subventricular and subgranular zones. Although cooling is a well-recognized therapy in cerebral ischemia, a role for RBM3 is largely unclear. 

The action of CIRP in ischemic brain injury is not well known. However, the level of CIRP mRNA decreases 3–6 h after transient ischemia in rat hippocampus and increases by five-fold in the cerebral cortex at 24 h after cerebral ischemia. However, no changes were seen after 48 h [90]. Apart from ischemia, hypothermia has been shown to considerably induce CIRP expression by approximately 30-fold until 24 h, and the combination of hypothermia and ischemia did not further enhance the CIRP level [91]. An increase of reactive oxygen species (ROS) is often the cause of oxidative stress during ischemia-reperfusion injury in the brain [92]. H_2_O_2_-induced ROS production is associated with down-regulation of CIRP expression levels [90]. The induction of endogenous or artificial overexpression of CIRP inhibits H_2_O_2_-induced apoptosis, indicating a neuroprotective role of CIRP [93,94]. Apart from the intracellular neuroprotective action of CIRP, the release of CIRP into the blood system can activate detrimental immune responses. For example, the secretion of CIRP from microglia after cerebral ischemia mediates TNF-α expression leading to neuroinflammation and causing neuronal damage both in vivo and in vitro [95]. Therefore, as long as CIRP is intracellularly localized, it protects neurons from apoptosis; but once CIRP is released from microglia, it mediates devastating neuroinflammation at the cellular level [96].

## 8. Hypothermia

The temperature regulatory response to systemic inflammation consists of hypothermia and the development of fever. Fever is a conserved evolutionary adaptive physiological response aimed at host survival [97], while hypothermia is maladaptive, associated with a poor clinical outcome [98,99]. The mortality rate in hypothermic sepsis patients is twice that of febrile patients [98,100], although the mechanisms for this detrimental effect of hypothermia is poorly understood. During regulated hypothermia, the core body temperature is lowered in response to a decrease in the thermoregulatory set-point, which evokes a variety of effector mechanisms that promote heat loss, diminishes heat production, and lowers core body temperature. Several species exhibits regulated hypothermia in response to food restriction [101], hypoglycemia [102,103], hypoxia [104,105], hemorrhage [106], dehydration [107], and infection [97,108,109]. The maintenance of normal body temperature, particularly under conditions of low ambient temperature, is always compensated by large energy loss. The adaptation to fever during infection is a highly energetic process and conserved throughout evolution [97], suggesting that the effects of high temperature on immune responses outweigh the high metabolic requirement. Induced hypothermia is used clinically for the treatment under the conditions of oxygen deprivation in cerebral ischemia, cardiopulmonary bypass surgery, and stroke [110,111]. The beneficial effect of therapeutic hypothermia under these conditions of oxygen deprivation appears obvious, but whether these conditions extend to inflammation condition is not yet known. The adaptive value of hypothermia could be achieved under a condition of energy depletion, which prevents the high energy cost of fever from host benefit. Hypothermia and fever represent extremes of a thermoregulatory continuum whose control is dependent on the metabolic capabilities of the host.

### 8.1. Therapeutic Hypothermia

**Figure 5:**
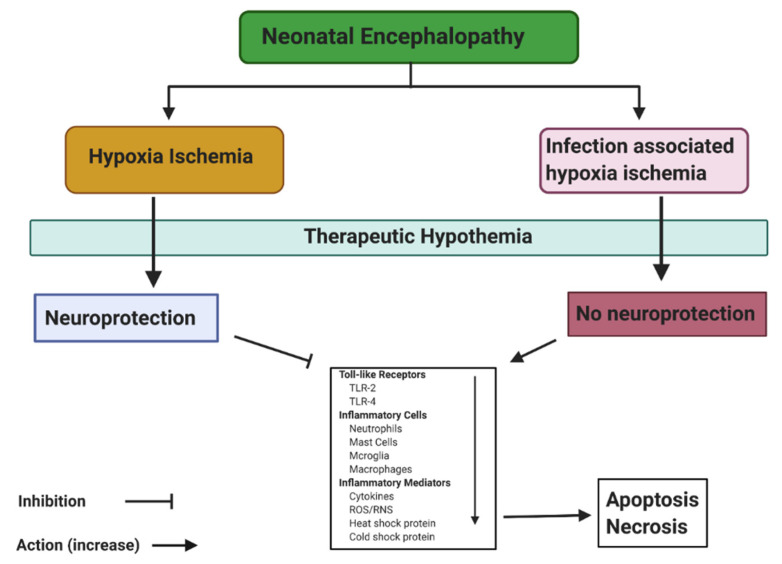
Inflammatory and cooling cascades following neonatal asphyxia. Dependent on the etiology of neonatal encephalopathy, therapeutic hypothermia can either be neuroprotective (following hypoxia-ischemia) or not neuroprotective (infection associated hypoxia ischemia) by inhibiting or activating different pathways leading to apoptosis or necrosis.

Therapeutic hypothermia is a neuroprotective therapy for neonatal HIE. Recently, therapeutic hypothermia has been recognized by the World Health Organization (WHO) as a contributing factor lowering morbidity and mortality risk in newborns [112,113,114]. Induced hypothermia is recommended at 33–34 °C for most of the therapeutic hypothermia practices [115]. Effects of therapeutic hypothermia include slowing down blood overload to the brain and reducing ATP consumption, including the retardation of destructive enzymatic reactions, suppression of free-radical reactions, protection of membranes fluidity, reduction of intracellular acidosis, inhibition of the biosynthesis, decrease of intracranial pressure [116,117,118], release and uptake of excitatory neurotransmitters [119,120,121], and depleted synapses, resulting in a reduction of brain activity, which results in less brain damage [122] (Figure 5). Therefore, induced mild-moderate therapeutic hypothermia provides the ability for tissues to endure anoxic no-flow states [123,124]. In this context, it has been estimated that for every 1 °C decrease in temperature, the cerebral metabolic rate decreases by 6–7% [125,126], and makes therapeutic hypothermia the most potent treatment at the moment to reduce ischemic brain injury by itself in HICs [127]. The question now is, is hypothermia neuroprotective following neonatal HIE in LMICs?

### 8.2. Accidental Hypothermia

Accidental hypothermia occurs when the core body temperature dropped to less than 35 °C, causing acute clinical risk. Accidental hypothermia requires invasive central rewarming interventions in order to prevent death [128]. There are common causes of accidental hypothermia, including cold-water immersion, environmental exposure, infection, metabolic conditions, drug-induced hypothermia, central nervous system (CNS) lesson, and malnutrition, but for the interest of this review, we focus on infection-induced hypothermia. Thermoregulatory responses to systemic inflammation are often regarded as maladaptive responses by the host. It has been shown that rodents regulate core body temperature during systemic inflammation with hypothermia by the selection of cool ambient temperatures and that this endogenous hypothermia is associated with enhanced survival [129]. Although the mechanisms regulating hypothermia are not fully understood, cytokines such as TNF-alpha, ILs, interferon (IFN)-gamma, and TRPV1 have been shown to induce or modulate hypothermia. TNF-alpha may function as a pro-endogenous cryogen, whereas IL-10 modulates TNF-alpha production and/or releases as a mechanism of hypothermia attenuation. IL-1beta and IL-6 are typically regarded as endogenous pyrogens but may regulate and/or induce hypothermia during viral and bacterial inflammation (Figure 5). A role for endogenous IFN-gamma in hypothermia has not been demonstrated, but the injection of this cytokine potentiates hypothermia through augmented production of other cytokines. It is clear that additional research is required in this area. Suggested areas for future research include a determination of the final mediator of hypothermia and its specific anatomical site of action, as well as the role of cytokines in the regulation of hypothermia under non-inflammatory conditions.

### 8.3. Accidental Hypothermia in the Context of Perinatal Asphyxia in LMIC

The studies conducted on the prevalence of neonatal accidental hypothermia due to non-warming in LMIC can not necessarily represent a standard hypothermia intervention. 

Previous hospital and community-based studies have examined case-fatality rates (CFRs) between those babies with and without accidental hypothermia and concluded that the risk of mortality is higher among those with hypothermia (Table 1). An analysis of 320 babies from a tertiary care facility in Recife, Brazil, indicated that moderate hypothermia (32.0–35.9 °C) on admission was an independent risk factor for neonatal death [130]. In the Islamic Republic of Iran, neonatal mortality was recorded high among babies with rectal temperatures less than 36.5 °C in the first 20 min after birth (8.8%) compared with normothermic babies (2.6%). However, these outcomes were not adjusted for weight and gestational age [131]. In Nigeria, an unadjusted case-fatality study showed a two-fold greater risk of mortality among the 62.0% of babies who were hypothermic upon admission [132]. In a community-based study in India, the case-fatality among 130 infants with hypothermia was estimated at 15.4%. Unfortunately, the study size was small (763 infants with only 20 deaths), and only a single fixed axillary temperature cutoff (35.0 °C) was used to classify infants. In Guinea-Bissau, it was recorded that infants with temperatures less than 34.5 °C were at five-times greater risk of mortality in the first week of life [133]. Population-based data from Nepal was used to examine the relationship between axillary temperature over the entire range of hypothermia values and mortality after the first temperature observed [134]. After adjusting for parameters like age, ambient temperature at measurement, sex, weight, gestational age, and ethnicity, mortality was increased by approximately 80% for every degree decrease in first observed axillary temperature. Mortality associated with accidental hypothermia was substantially greater among preterm infants [134].

## 9. Future Direction

The impact of infection on hypothermia outcome and the role of HSPs and CSPs in response to hyperthermia and hypothermia have not been studied extensively. In the currently available data, there is controversy whether TRPs regulate hypothermia, although TRPs are known to function as an endogenous thermal sensor. Unfortunately, only some data are available on the effects of endogenous TRPs on temperature regulation. However, this data is required to delineate an endogenous role of any TRPs on thermoregulation. Figure 4 provides a temperature range model of TRPs temperature interaction that mediates hyperthermia in response to an inflammatory stimulus. Our current understanding of COX-2-stimulating-PGE2-induced hyperthermia and the mediation of TRPs is discussed in this review. 

However, there are accumulated data on the role of cytokines in both hyperthermia and hypothermia; huge gaps still exist in the current field of research. Following proposed studies for future knowledge might include the determination of final mediators of hypothermia and hyperthermia. Several studies have examined COX-2 and prostaglandins as the inducer of hyperthermia, but these results have been contradictory. TRPs levels were reported to increase during LPS-induced hyperthermia in rats [137], while COX-2 inhibitors, such as non-steroidal anti-inflammatory drugs (NSAIDs), attenuate, exacerbate, or have no effect on hyperthermia. Thus more studies are required to clarify the final mediators involved as well as specific brain areas involved in the regulation of this response.

Determination of cytokine effects during infectious and non-infectious conditions on hypothermia and hyperthermia using a specific cytokine antagonist, as this might have a direct effect on the thermal set-point.

Finally, the majority of studies on cytokines and hypothermia have been performed in rodents. The exact cytokine effects in humans during therapeutic hypothermia are not fully understood. Given the large difference in surface area to body mass ratio between rodents and humans and the reduced sensitivity of mice and rats to bacterial products such as LPS (i.e., much larger doses are required to induce fever in rodents), extrapolation between species is difficult. Furthermore, there is the concern that the thermoregulatory actions of a cytokine may be beneficial, whereas other physiological effects may be harmful, thus complicating our assessment of potential beneficial treatment effects. Only through careful analysis of cytokine action in several models of systemic inflammation, with attention to hypothermia as well as fever, can these obstacles be identified and overcome.

## 10. Conclusions

In this present review, we discussed how heat shock and cold shock proteins’ responses to hyperthermia and hypothermia might be used to better understand the molecular pathways in the face of infection and hypoxic-ischemic injury. Both HSPs and CSPs exert immunomodulatory functions in the mobilization of immune cells. It is apparent that a better understanding of the complex interaction between the HS and CS responses and the inflammatory pathways is critical for infection, sepsis, and inflammation.

## Figures and Tables

**Table 1:** Selected Hospital/community association with hypothermia and mortality risk in the low- and middle-income countries (LMICs).

Location	No. of Patients	Mortality Outcomes	Author, Year
Bissau, Guinea-Bissau	2926	- Adjusted for weight, temperatures <34.5 °C were associated with mortality 4.81 (95% CI: 2.90–8.00) times greater in the first seven days of life - Hypothermia-associated mortality risk was elevated through two months of life	Sodemann, 2008 [133]
Sagamu, Nigeria	150	Unadjusted fatality rate among hypothermic infants was 2.26 (95% CI: 1.14–4.48) greater than normothermic infants	Ogunlesi, 2008 [132]
Sarlahi, Nepal	23,240	- Adjusted mortality risk increased 80% for every 1 °C decrease in first observed axillary temperature decrease - Adjusted mortality risk was 6.11 (95% CI: 3.98–9.38) times higher among infants <35.0 °C. Preterm babies at higher risk of hypothermia-associated mortality	Mullany, 2010 [134]
Recife, Brazil	320	- Adjusted odds of death among babies with hypothermia had a odds ratio of 3.49 (95% CI: 3.18–3.8)	da Mota Silveira, 2003 [130]
Gadchiroli, India	763	- Case-fatality of hypothermia was 15.4%, and was significantly greater than those without hypothermia	Bang, 2005 [135]
Tehran, Iran	900	- Unadjusted fatality was 8.8% among hypothermic infants compared with 2.6% among normothermic	Zayeri, 2007 [136]

## Data Availability

Not applicable.
